# Calorimetry informed visual digital model for continuous flow photobromination

**DOI:** 10.1038/s42004-026-02023-5

**Published:** 2026-04-11

**Authors:** Yiming Xu, Yun Zou, Junfei Zhang, Fujun Li, Shengyang Tao

**Affiliations:** State Key Laboratory of Fine Chemicals, Frontier Science Center for Smart Materials, School of Chemistry, Dalian University of Technology, Dalian, China

**Keywords:** Photocatalysis, Flow chemistry, Reaction mechanisms

## Abstract

Calorimetry data for continuous-flow photochemistry are scarce, hindering kinetic model development and safe scale-up. Here we show that in situ calorimetry combined with designed experiments enables a quantitative, heat-consistent kinetic description for the continuous-flow photobromination of (*E*)-methyl 2-(methoxyimino)-2-(*o*-tolyl) acetate (EMMA). We record total heat release, conversion, and selectivity over defined windows of residence time and light intensity, and fit a two-step kinetic scheme constrained by the calorimetric heat-release data. The model predicts total heat with *R*^2^ = 0.957 and conversion with *R*^2^ = 0.894, while the second bromination step shows negligible dependence on light intensity within the explored window, indicating thermally driven behaviour. We translate these parameters into a real-time visual digital model that maps the spatial distributions of conversion, selectivity, and heat-release rate and provides thermal-management guidance in real time. This approach extends the calorimetric dimension of process analytics for photochemistry and supports safer, more efficient process development.

## Abstract (web summary)

Calorimetry data for continuous-flow photochemistry are scarce, hindering kinetic model development and safe scale-up. Here, the authors combine in situ calorimetry with design of experiments to build a quantitative, heat-consistent kinetic model and a calorimetry-informed visual digital model for the continuous-flow photobromination of (*E*)-methyl 2-(methoxyimino)-2-(o-tolyl)acetate, enabling real-time thermal-management guidance and extending the calorimetric dimension of process analytics.

## Introduction

Over the past decade, the use of photochemistry in synthetic processes has expanded rapidly. As light sources and reactor technologies have improved by leaps and bounds, photochemical reactions are rapidly approaching a scalable and highly controllable processing regime^1–5^. Owing to the high surface-to-volume ratio, microreactors offer superior heat-transfer performance and precise light delivery, thereby improving irradiation uniformity, mitigating over-irradiation, and providing a practical path from millilitre- to kilogram-scale production^6–9^. However, achieving deep control over photocatalytic kinetics and reaction progress remains challenging. The calorimetric and kinetic data sets needed to underpin scale-up and safety assessments remain scarce, which constrains mechanistic verification and engineering predictability^9,10^.

In photochemical halogenation, bromination is a strongly exothermic reaction that combines high atom economy with broad functional-group tolerance. Under visible-light irradiation, Br₂ undergoes homolytic cleavage across its broad, featureless 300–750 nm, thereby releasing bromine radicals that propagate chain reactions^11–13^. Leveraging this photochemical behaviour, we employ 405 nm LED irradiation and generate liquid bromine in situ by continuously reacting hydrobromic acid with hydrogen peroxide, which serves as the bromine source for photobromination. We selected (*E*)-methyl 2-(methoxyimino)-2-(*o*-tolyl) acetate (EMMA) as the substrate, because its monobrominated product is a key intermediate in agrochemical synthesis^14–16^. When carried out in a microreactor, this highly exothermic photobromination allows precise control over the residence time and reaction temperature. However, real-time, quantitative access to the exotherm, substrate conversion, and product selectivity still relies on in situ microscale calorimetry coupled with kinetic analysis^17,18^.

Reaction calorimetry plays a central role in process-safety assessment and kinetic modelling. However, direct translation of conventional batch calorimetry data to microreactors is not valid because it fails to capture the markedly different physical parameters, including mixing, mass- and heat-transfer, and residence time distribution, that govern continuous-flow systems^19,20^. In photochemical systems, flow calorimetry faces additional obstacles, including the effective coupling of the calorimeter to the light field and the separation of chemical heat release from photothermal heating^21,22^. These issues have hampered the accurate determination of heat release in continuous photochemical processes^23–26^. To address these limitations, we previously developed a Dynamic Tracking Referenced Continuous Calorimeter (DTRCC), inspired by biological thermoregulation, which measures reaction exotherms rapidly and accurately under nonadiabatic continuous-flow conditions^27^. When applied to photobromination, DTRCC captures the time-resolved evolution of the reactor-level heat-release signal in real time, a capability that is difficult to achieve with traditional methods.

Here, we integrate in situ flow calorimetry with design-of-experiments methodology and kinetic modelling to efficiently obtain key data and elucidate the reaction kinetics. Using a D‑optimal design-of-experiments, we systematically select reaction conditions spanning broad ranges of temperature, residence time, and irradiance (optical power density), while minimising the number of experiments^28–31^. Under each set of conditions, the DTRCC continuously records the exotherm, whereas conversion (*X*) and monobromide selectivity (*S*) are determined independently by ex situ high-performance liquid chromatography (HPLC) analysis of quenched steady-state outlet samples. Importantly, *X* and *S* are not inferred from calorimetry in the D-optimal experimental campaign. Instead, *Q* (calorimetry) and *X*/*S* (HPLC) constitute three independently measured responses used for subsequent model identification and validation. We then construct a kinetic model that uses temperature, residence time, and irradiance as independent variables to quantify their effects on reaction rates and selectivity.

In this work, we address a key methodological gap in the integration of photochemistry, flow chemistry, calorimetry, and kinetics, thereby enabling the acquisition of critical data that were previously inaccessible. Process Analytical Technology (PAT) advocates the integration of in situ sensors, such as ultraviolet–visible (UV–Vis), Raman, infrared (IR), and nuclear magnetic resonance (NMR), with reactors to enable real-time monitoring and model updating^26,32^. By contrast, calorimetry for photochemical processes remains an underdeveloped dimension within PAT. We correlate the measured exotherm data with independently measured conversion and selectivity and map these datasets onto the microreactor geometry to generate a visual digital model of the reaction process. After the kinetic model and reaction enthalpies have been established using the calorimetry/HPLC dataset, the subsequent online stage no longer requires concurrent HPLC analysis; instead, the measured heat signal is assimilated together with the identified reaction enthalpies to refine the predicted selectivity. This model enhances our understanding of the coupled heat, mass, and light fields and provides practical support for real-time monitoring and optimisation of photoreactors.

Accordingly, we carry out the visible‑light photobromination of EMMA at 405 nm in a microreactor. Using the DTRCC, we quantify the reactor-level heat-release signal in situ under varied conditions and, through a D-optimal design, efficiently collect data on total heat release, substrate conversion, and selectivity for the monobromide product. On this basis, we develop a kinetic model that uses temperature, residence time, and irradiance as inputs, and we further construct a calorimetry-informed visual digital model that predicts and renders three-dimensional distributions of heat-release rate, substrate conversion, and product selectivity within the reactor. Implemented on the calorimetry platform, this integrated workflow delivers a traceable, visual, and predictive description of thermokinetic coupling in continuous photochemistry and provides essential data for safe scale‑up and process optimisation^27,33^. This integrated workflow enables a quantitative kinetic study of photobromination using in situ microflow calorimetry together with real-time visualisation of the process.

**Fig. 1:**
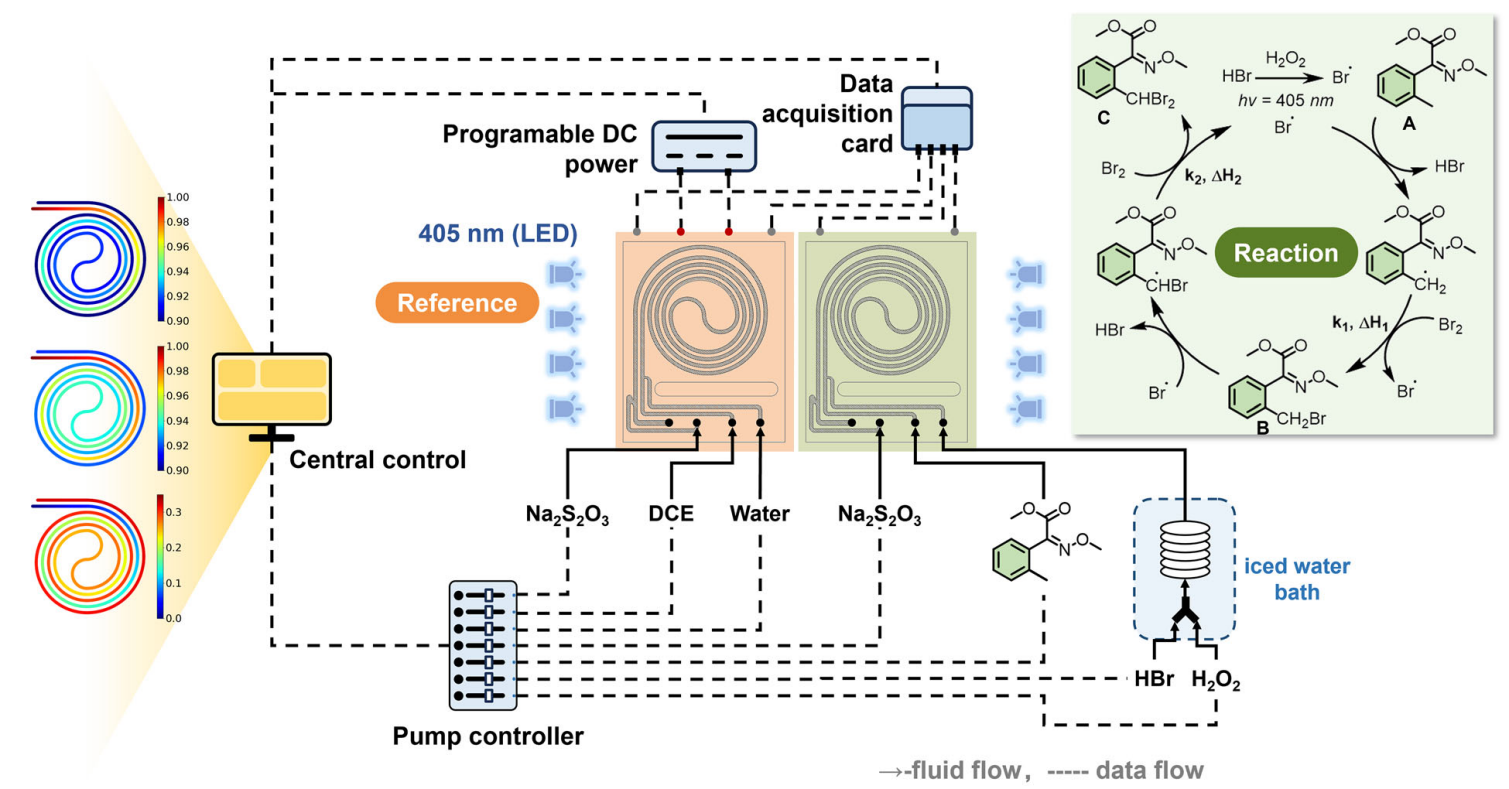
Calorimetry-informed visual digital model for continuous flow photobromination. The figure summarises the integrated experimental platform, including continuous bromine generation, the paired test and reference DTRCC modules under 405 nm irradiation, the control/data-acquisition architecture, representative reactor-coordinate maps, and the two-step reaction scheme used for model construction.

Fig. 1 schematically summarises the integrated experimental platform and the end-to-end data workflow used in this study. Briefly, bromine is generated continuously from HBr and H_2_O_2_ in a precooled coil and merged with an EMMA solution prior to entering the test microreactor. The DTRCC comprises a test module and a reference tracking module that share the same microreactor architecture and 405 nm irradiation conditions. Each module integrates a glass microreactor chip thermally coupled to a Seebeck element, while a programmable DC power supply dynamically adjusts the reference thin-film heater to match the Seebeck signal of the test module, yielding the steady-state reaction heat-release power *P*. The total heat release reported in this work is obtained as *Q* = *Pτ* for a given residence time *τ*. In parallel, steady-state outlet samples are quenched and analysed by ex situ HPLC to quantify conversion and selectivity, providing independent composition references for model identification, validation, and calorimetry-informed online visualisation.

## Results

### D-optimal experimental design for photobromination

To obtain high-quality data for mechanism elucidation and kinetic modelling within a limited number of experiments, we adopted a D-optimal experimental design to efficiently sample a three-factor design space. The experimental platform and the associated control and data-acquisition workflow are schematically shown in Fig. 1 and described in Methods. The factors considered were reaction temperature (30–45 °C), residence time (40–120 s), and 405 nm irradiance (9.1–49.2 mW·cm^-2^). For selecting the D‑optimal points, we used a full quadratic response‑surface as the candidate model (3 main effects, 3 squared terms, and 3 two‑factor interactions plus an intercept, 10 basis functions in total). After data collection, we revisited the regression according to the parsimony principle and performed hierarchical term screening. Non‑significant interaction or quadratic terms were removed where appropriate, and the reduced surrogate models are reported in Supplementary Note 6. Importantly, this response‑surface model is used as an empirical surrogate for design and visualisation, whereas mechanistic interpretation and transferable prediction rely on the subsequent kinetic model. To reduce the condition number of the information matrix and enhance comparability and interpretability of the coefficients, each predictor was centred and coded to a dimensionless variable. A candidate set was generated within the above factor ranges (see Supplementary Note 1).

By employing a D-optimal design, we conducted a resource-efficient experimental campaign comprising only 36 runs, which satisfied all requirements for parameter estimation and model diagnostics and reduced the experimental burden by 55% compared with a full-factorial design (80 runs). Five replicate experiments at the centre point (40 °C, 80 s, 35.0 mW·cm^−^^2^) were performed to estimate pure error and to monitor platform stability.

**Fig. 2:**
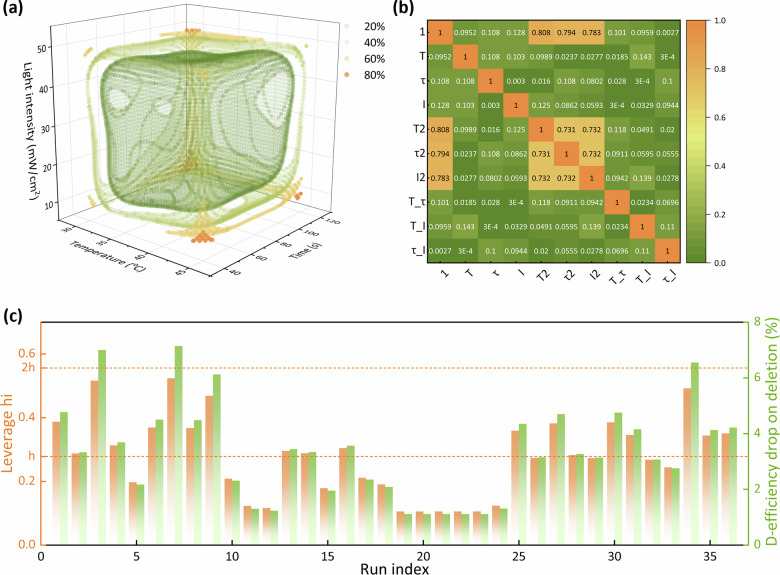
D-optimal design for photobromination: coverage of the design space and robustness diagnostics. **a** Composite three-dimensional isosurfaces of prediction variance across the three-factor space. **b** Aliasing/correlation heat map of the design matrix. **c** LOO diagnostics: leverage values and reductions in D-efficiency.

As indicated by the three-dimensional prediction-variance isosurfaces in Fig. 2a, the model exhibits fairly uniform prediction variance across the operable region, with only local increases near the boundaries and vertices of the design space. This pattern suggests comparable prediction precision over most of the region, corresponding to approximately the 80% confidence level (see Supplementary Note 2). The correlation heat map of the design matrix (Fig. 2b) shows that most parameter columns have low absolute pairwise correlations. Although moderate correlations appear among the squared terms, a common feature of quadratic models, these do not hinder the identification and estimation of main and interaction effects. The leave-one-out (LOO) diagnostics (Fig. 2c) show leverage values for all runs below the empirical threshold of 2 *h*, where *h* = *p*/*n* = 10/36 ≈ 0.28). Deleting any single run reduces the design’s D-efficiency by < 8% (see Supplementary Note 3). Hence, no single data point unduly dominates the model fit.

Benefiting from the rapid response of the DTRCC system and the high-information density afforded by the D-optimal design, we obtained a comprehensive and complementary data set from only 36 runs, providing a reliable basis for subsequent kinetic parameter estimation and mechanistic discrimination.

### Effects of reaction conditions and their interactions

**Fig. 3:**
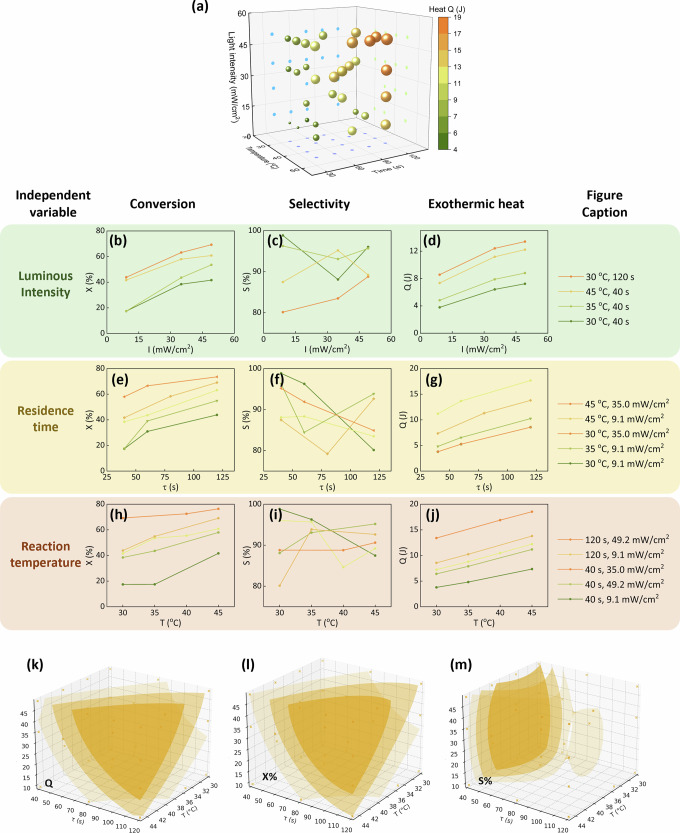
Effects of temperature, residence time, and irradiance on conversion, monobromide selectivity, and total heat release. **a** 3D bubble chart with axes for temperature, residence time, and irradiance; bubble colour encodes Q (total heat release) and bubble size encodes X. **b**–**d** Main-effect plots for irradiance. **e**–**g** Main-effect plots for residence time. **h**–**j** Main-effect plots for temperature. **k**–**m** Three-dimensional isosurfaces for X, S, and Q constructed from the experimental data.

To delineate how operating variables shape the reaction outcome, we systematically examined the effects of temperature, irradiance, and residence time on conversion (*X*), selectivity for the monobrominated product (*S*), and total heat release (*Q*) (see Supplementary Note 4). Here, *Q* was obtained in situ from the DTRCC, whereas *X* and *S* were calculated from concentrations measured by offline HPLC analysis of quenched outlet samples. The DTRCC provides the reactor-level heat-release power (heat-flow rate) integrated over the full microreactor; thus, *Q* is an integral metric and does not resolve axial heat-release rate directly. Figure 3 summarises the main and interaction effects of these factors. For conversion (*X*) and total heat release (*Q*), the quadratic surrogate provides an excellent fit (*R*² > 0.95; Supplementary Note 5). In contrast, the selectivity (*S*) varies only weakly within the investigated window, and the quadratic surrogate explains only a limited fraction of its variance (*R*^2^ ≈ 0.285). Therefore, the *S* isosurface is presented for qualitative guidance only. Consistent with the parsimony principle, only statistically supported terms are retained and discussed in the reduced models (Supplementary Note 6).

Temperature and residence time are the primary determinants of *X* and *Q*, with temperature exerting the stronger effect. Increasing the temperature from 30 °C to 45 °C raises *X* from approximately 40% to about 70%, and *Q* increases in concert (Fig. 3h, 3j). Extending the residence time from 45 s to 120 s also increases *X* and leads to greater total heat release (Fig. 3e, 3g). These factors act synergistically. At higher temperatures, increasing the residence time can push *X* towards 80% and drive *Q* to its maximum (Fig. 3k, 3m). At low temperatures, even long residence times yield only modest gains, and *Q* remains at a low level, indicating that the reaction is limited by temperature. Within the investigated irradiance window of 9.1–49.2 mW·cm^-2^, irradiance exerts a positive but smaller effect on *X* and *Q* (Fig. 3b, 3d). Increasing irradiance accelerates the reaction, modestly elevates *X*, and slightly increases *Q*, yet its impact remains weaker than that of temperature and residence time. The trends in *X* and *Q* are similar across irradiance levels, and no strong interaction with irradiance is evident. The selectivity *S* remains high, typically above 90% under most conditions, with only slight declines at extreme temperatures or long residence times (Fig. 3c, 3f, 3i, 3l). Model diagnostics corroborate these observations. The main factors contributing to *X* and *Q* are statistically significant (*p* <  0.05), the overall fit quality is high (*R*² > 0.95), and prediction errors are small, with a low root mean square error (RMSE). The remaining interaction terms are comparatively minor (see Supplementary Note 6).

The three-dimensional isosurfaces for *Q* and *X* (Fig. 3k, 3l) are concentrated in regions where both temperature and residence time are large, and these regions expand at higher irradiance, *I*. Hence, substantially increasing *Q* and *X* requires elevating the temperature and the residence time simultaneously. As the conditions become more extreme, the spacing between successive high-response shells narrows, signalling diminishing returns. When the temperature lies in the range of about 40–45 °C or the residence time exceeds about 100 s, further increases provide only marginal additional gains. By contrast, the isosurface for *S* (Fig.3m) shows a different distribution, with higher values of *S* under milder conditions, consistent with suppression of side reactions at lower temperature. Because the quadratic model for *S* explains only a small fraction of the variance (*R*^2^ ≈ 0.285), Fig. 3m should therefore be viewed as qualitative guidance.

The analysis reveals that all three factors exhibit not only pronounced main effects but also measurable interaction effects. The close alignment between the calorimetric signal and conversion underscores the value of in situ calorimetry as a process-analytical tool, as it enables quantitative tracking of reaction progress and supports the identification of an optimal operating window. These data provide a robust basis for the subsequent kinetic modelling and parameter estimation.

### Kinetic model development

Guided by the above experiments and the reaction scheme shown in Fig. 1, we modelled the process as a two-step consecutive bromination sequence (A → B → C), where EMMA is denoted as A, the monobrominated intermediate as B, and the dibrominated product as C. In the first step, A undergoes monobromination to form B, and in the second step, B is further brominated to form C. Bromine radicals generated by light irradiation supply the radical flux required for initiation and propagation. The reactor is treated as a plug-flow reactor (PFR), and the set temperature is taken as the reaction temperature. To quantify the influence of light on each step, we introduced an irradiance exponent (*m*) into the kinetic model to represent the dependence on light intensity. Based on this mechanism, the kinetic differential equations for the relevant species can be written as

1 $$\frac{d\left[A\right]}{{dt}}=-{k}_{1}{I}^{{m}_{1}}[A]$$

2 $$\frac{d\left[B\right]}{{dt}}={k}_{1}{I}^{{m}_{1}}[A]-{k}_{2}{I}^{{m}_{2}}[B]$$

3 $$\frac{d\left[C\right]}{{dt}}={k}_{2}{I}^{{m}_{2}}[B]$$

Here $\left[A\right]$, $\left[B\right]$ and $\left[C\right]$ denote the molar concentrations of EMMA (A), monobromide (B), and dibromide (C), respectively (mol·L^−^^1^). The parameters ${k}_{1}$ and ${k}_{2}$ are the apparent rate constants for the first and second steps (s^-1^). $I$ is the light intensity (mW·cm^−^^2^), and ${m}_{1}$ and ${m}_{2}$ are the corresponding irradiance exponents. The dependence of the apparent rate constants on light intensity and temperature is described by an Arrhenius-type relation:

4 $${k}_{i}(T,I)={A}_{i}{I}^{{m}_{i}}\exp (-{E}_{i}/{RT})$$

where ${A}_{i}$ is the pre-exponential factor with units of s^−^^1^·(mW·cm^−2^)^{−*m*_*i*_} (with *I* expressed in mW·cm^−2^), and ${E}_{i}$ is the apparent activation energy (J·mol⁻¹). Fitting this model to experimental data yields the following parameter values for the first and second steps: *A*_1_ = 77.36, *m*_1_ = 0.381, *E*_1_ = 26.0 kJ·mol^−1^, *A*_2_ = 3.42 × 10^−3^, *m*_2_ = 0.064, *E*_2_ = 1.7 kJ·mol^−1^. The corresponding 95% confidence intervals and parameter correlation matrices are reported in Supplementary Tables 10–12. Notably, the pre-exponential factors and activation energies are strongly correlated in the Arrhenius form, and we therefore also report reference rate constants at a representative temperature and irradiance. The fitted parameters indicate a sub-linear irradiance response and moderate activation energies, which are consistent with expectations for a light-initiated radical mechanism (see Supplementary Note 7).

The calorimetry-consistent heat-release model is written as

5 $$Q={n}_{A}\left[-\Delta {H}_{1}X-\Delta {H}_{2}X\left(1-{S}_{1}\right)\right]$$

**Fig. 4:**
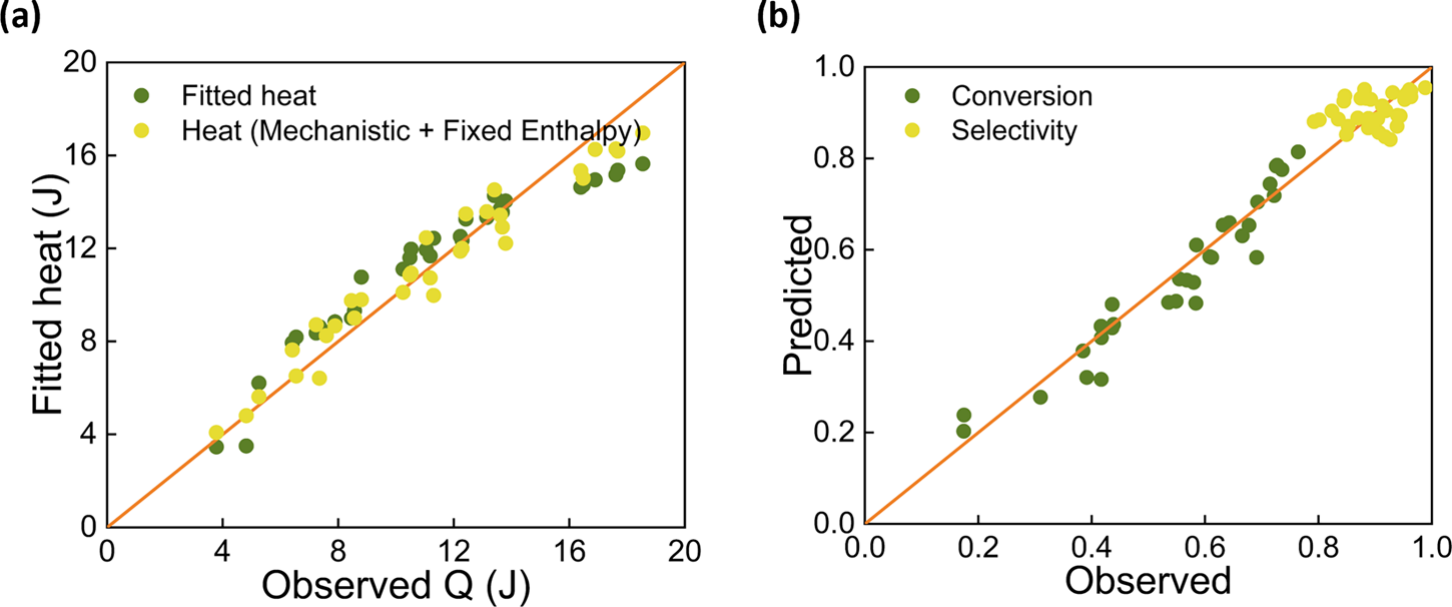
Model–experiment comparisons. **a** Measured total heat versus model prediction. **b** Measured conversion and monobromide selectivity versus model prediction.

From the regression of the model against the experimental data, the reaction enthalpies are Δ*H*_1_ = −293.9 kJ·mol^−1^ and Δ*H*_2_ = − 113.8 kJ·mol^−1^. Comparable calorimetric enthalpy data for photochemical benzylic brominations are scarce. However, Steiner et al. quantified the heat effects of related benzylic bromination chemistry by isothermal titration microcalorimetry at 25 °C and reported exotherms on the order of 10^2 ^kJ·mol^−1^ (including an estimated ~−63 kJ·mol^−1^ contribution from the benzylic substitution step)^34^. Notably, those microcalorimetry data were acquired without concurrent irradiation and therefore do not include any additional irradiation-associated thermal load. Accordingly, Δ*H*_1_ and Δ*H*_2_ are interpreted here as effective (apparent) enthalpies specific to the present irradiated flow system and two-step reaction network, extracted by reconciling the in situ calorimetric signal with the kinetic model. The predicted total heat agrees well with the calorimetric measurements, with *R*^2^ = 0.957, indicating self-consistency between the thermal and kinetic descriptions in this system (Fig. 4a). The fit for conversion is also of high quality, with *R*^2^ = 0.894 (Fig. 4b). The mean absolute error (MAE) for the selectivity of the monobrominated product is about 4.15%. In addition, the kinetic model was validated at the composition level by comparing predicted outlet concentrations of A, B, and C with ex situ HPLC measurements (Supplementary Tables 8, 9), showing MAE values of 0.026, 0.021, and 0.012 mol·L^−1^ for A, B, and C, respectively.

Notably, within the present experimental window, the second step shows no statistically significant dependence on light intensity, indicating that this step is not primarily controlled by photoexcitation. Once the intermediate B is formed, its subsequent conversion is governed primarily by its own kinetics, and increasing light intensity does not accelerate this step. This behaviour suggests that the second step may proceed predominantly through a thermally induced or spontaneous pathway. In line with this weak sensitivity, the fitted E2 is small, indicating only a mild temperature dependence of step 2 over 30–45 °C.

In summary, the coupled model combining light-initiated and thermally governed rates consistently captures the observed trends in conversion and total heat release. The methodology is readily transferable to other photochemical systems and is well suited to in situ calorimetry for online prediction and process control.

### Calorimetry-informed visual digital model for continuous-flow photobromination

**Fig. 5:**
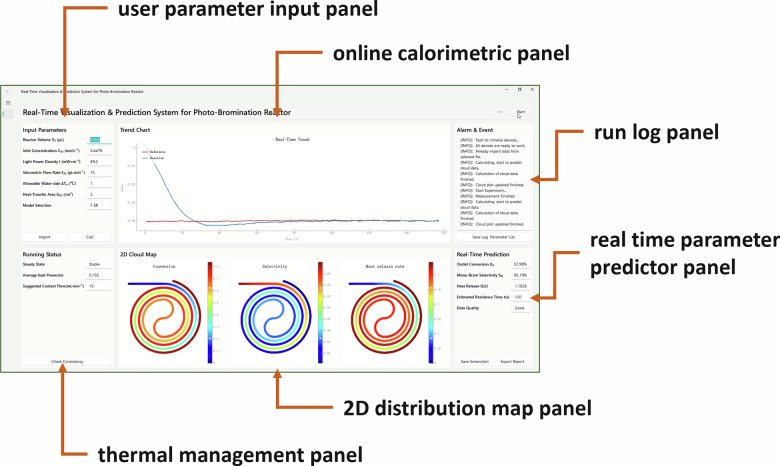
Real-time interface of the calorimetry-informed visual digital model. The interface comprises a user-parameter input panel, an online calorimetry panel, a real-time parameter-predictor panel, a two-dimensional distribution-map panel, a thermal-management panel, and a run-log panel.

We developed and implemented a visual digital model driven by in situ continuous-flow calorimetry that unifies the heat-release curve, spatial distribution, and thermal management within a single workflow. The key element is a model-based data-assimilation step that reconciles a kinetics-based prior with the measured calorimetric heat-release signal to produce a heat-consistent posterior prediction. Because the DTRCC yields an integral heat-release signal for a given operating point, location-specific heat-release rate maps are obtained computationally by mapping residence time to reactor coordinates under a plug-flow assumption and by integrating the kinetics-based local heat-release rate (Supplementary Note 8). The interface is shown in Fig.5, and the full operating procedure is provided in the Supplementary Movie 1. The model integrates six modules: a user parameter input panel, an online calorimetry panel, a real-time parameter-predictor panel, a two-dimensional distribution-map panel, a thermal-management panel, and a run-log panel. The predictor displays conversion, selectivity for the monobrominated product and heat-release rate under the current conditions, and the two-dimensional map presents their spatial distributions. The thermal-management panel recommends inlet-temperature ranges and coolant flow rates. The run-log records, with timestamps, each parameter change, steady-state determination, assimilation update, and check result, thereby facilitating traceability.

The system first constructs a kinetics-based prior prediction from the input parameters and subsequently computes a posterior prediction calibrated against the experimental calorimetric curve. The user enters the relevant parameters through the interface, which are combined with the kinetic model to generate prior predictions, followed by the calorimetry stage. After obtaining the measured heat data, the reaction enthalpy is used to update and refine the predicted selectivity distribution. In this step, conversion is retained from the kinetics-based prior, whereas selectivity is corrected by matching the measured heat signal using Δ*H*_1_ and Δ*H*_2_. Thus, once the model has been established, the online visualisation relies on the calorimetric signal rather than concurrent chromatographic measurements. A visual digital model of the photobromination process is then constructed on the basis of these predictions. The detailed workflow is provided in Supplementary Note 8.

Thermal-management recommendations are driven directly by the online heat-release power. For jacketed water cooling and a specified allowable temperature rise (Δ*T*_*w*,allow_) on the water side, the required mass flow rate of cooling water is given by

6 $${m}_{w}(t)=\frac{Q\left(t\right)}{{c}_{p,w}\,\Delta {T}_{w,{{{\rm{allow}}}}}}$$

Here ${c}_{p,w}$ is the specific heat capacity of water (J·kg^−1^·K^−1^). Consistency is checked using the log-mean temperature-difference (LMTD) method together with an independently calibrated overall heat-transfer coefficient (*U*) (W·m^−2^·K^−1^) and heat-exchange area (*A*) (m^2^).

7 $$P={UA}\Delta {T}_{{lm}}$$

8 $$\Delta {T}_{{lm}}=\frac{\Delta {T}_{{{{\rm{in}}}}}-{\Delta T}_{{{{\rm{out}}}}}}{{{{\mathrm{ln}}}}\left(\Delta {T}_{{{{\rm{in}}}}}/\Delta {T}_{{{{\rm{out}}}}}\right)}$$

Here, ${T}_{{{{\rm{in}}}}}$ and ${T}_{{{{\rm{out}}}}}$ denote the inlet and outlet temperatures of the cooling water, respectively.

This visual digital model couples the kinetic model with in situ calorimetry within a common computational framework. It retains the generality of the mechanistic model while rapidly adapting to changing operating conditions through assimilation of the heat signal. Importantly, because the calorimetric signal is measured in situ for each operating point, it provides a real-time consistency check between model prediction and actual reactor behaviour and is therefore not redundant even when a kinetic model is available for offline simulation. Compared with prediction based solely on kinetics, the incorporation of real-time calorimetry enables more accurate and timely identification of heat-release-rate hot spots and provides cooling-strategy recommendations, offering a practical tool for process safety and robust operation in highly exothermic photochemical systems.

## Discussion

By leveraging in situ continuous-flow calorimetry with the DTRCC, we obtain a multidimensional description of continuous-flow photobromination. In contrast to strategies that rely solely on concentration measurements or spectroscopic probes, the real-time heat signal provides a physical anchor tightly coupled to reaction progress, thereby improving parameter identifiability and the robustness of extrapolation. Under 405 nm irradiation, temperature and residence time jointly control EMMA conversion and total heat. Their synergistic effect is most pronounced at high temperatures (*T*) and long residence times (*τ*). Light intensity increases the rate but exhibits a sub-linear response and diminishing returns under extreme conditions, which is consistent with qualitative expectations for radical-chain processes in which, beyond initiation, the propagation and termination steps, as well as absorption depth and local mass transfer, govern overall performance.

The two-step kinetic scheme unifies heat release, conversion, and selectivity within a single parameter set. The reaction enthalpies indicate that the first step dominates the overall exotherm, which explains the close alignment between conversion and heat across the design space. Furthermore, the model’s reproduction of total heat approaches the experimental uncertainty. The fits for conversion remain strong, and the mean absolute error in selectivity is within a few percentage points, providing evidence for the coherence and practical utility of the heat-constrained kinetic description. Importantly, the second step shows no significant dependence on light intensity within the present experimental window, suggesting a thermally driven or light-independent pathway. This finding has implications for multistep photohalogenations, in which differentiated light and thermal control across steps may sustain high selectivity while avoiding ineffective light-power input.

From a resource-efficiency perspective, the D-optimal design reduces experimental burden without sacrificing identifiability, as the prediction variance remains largely uniform over the working region and no single run unduly influences the fit. The DTRCC overcomes typical hurdles in coupling calorimetry with irradiation, enabling reliable separation and quantification of chemical heat under nonadiabatic, illuminated flow conditions. Taken together, these features increase both the statistical efficiency of model training and the overall sensitivity and speed of safety evaluation.

While a fixed kinetic or process model can be used offline for in silico optimisation, it cannot indicate in real time whether the operating reactor continues to follow that model. For engineering practice, the digital model projects the core information on heat, mass, and light fields into reactor coordinates and uses calorimetry for online correction so that prior kinetics and the measured heat behaviour remain consistent. When the operating parameters change, it rapidly updates the spatial distributions and cooling recommendations, converting potential hot spots, under-reacted zones, and heat-exchange limitations into actionable guidance on the operating window, thereby adding a critical calorimetric complement to photochemical PAT. Beyond the UV–Vis or Raman dimensions, calorimetry provides a safety-relevant metric that can be integrated with automated control to close the loop between measurement, modelling, and control.

There are clear scope boundaries. The kinetic and digital models assume plug-flow behaviour and a two-dimensional equivalent residence-time field, neglect radial concentration and temperature gradients, and treat the light field as a uniform irradiance. These approximations are reasonable for microchannels with efficient heat removal but merit re-evaluation for larger hydraulic diameters, non-uniform illumination, or higher heat-release rates. The statistical model for selectivity has limited explanatory power for the present data set, likely reflecting the small dynamic range of *S*, measurement noise, and the presence of multiple side pathways. Radiative transport and spatial absorption were not explicitly coupled. For strongly absorbing or scattering media, the photon-flux distribution may become rate-limiting. Nevertheless, the calorimetry-centred, data-integrated paradigm established here should generalise to other exothermic photochemical processes, particularly where safety-constrained operating-window identification and scale-up are essential. With richer in situ spectroscopy measurements, radiative-transfer calculations, and three-dimensional heat-exchange simulations, transferability across equipment and scales is expected to further improve.

## Methods

### Materials and reagents

(*E*)-methyl 2-(methoxyimino)-2-(*o*-tolyl)acetate (EMMA, AR, 95%) was obtained from Zhengzhou Alfa Chemical Co., Ltd. HBr (ACS, 48%) and H_2_O_2_ (AR) were purchased from Aladdin. Na_2_S_2_O_3_ (AR), anhydrous sodium sulfate (AR), and acetonitrile (HPLC grade) were obtained from Tianjin Kemiou Chemical Reagent Co., Ltd. 1,2-dichloroethane (DCE, AR) was obtained from Damao Chemical Reagent Factory. All reagents were used as received, and deionised water was used throughout.

### Light source and reactor setup

A 405 nm light-emitting diode (LED) light source (LumiDox II) was used. To deliver uniform irradiation to both test and reference modules, we 3D-printed a custom white polylactic acid (PLA) holder using a Raise3D Pro2 Plus printer, ensuring identical light intensities on both modules.

### Dynamic Tracking Referenced Continuous Calorimeter

Calorimetry was performed using a Dynamic Tracking Referenced Continuous Calorimeter (DTRCC). The DTRCC operates as a nonadiabatic, power-compensation heat-flow calorimeter and comprises a test module and a reference tracking module that share the same microreactor architecture and irradiation conditions. Each module integrates a glass microreactor chip coupled to a Seebeck element that converts the heat-flow rate into a voltage signal. During measurements, a thin-film heater in the reference module is dynamically adjusted to match the Seebeck signal of the test module, thereby cancelling environmental and photothermal disturbances. Under steady state, the electrical compensation power required for signal matching equals the reaction heat-release power generated in the test microreactor. In this work, the reported total heat release *Q* is obtained from the steady-state heat-release power *P* as *Q* = *P τ*, corresponding to the integral heat released by the reaction stream over the residence time τ in the microreactor.

### Photobromination procedure

Bromine (Br_2_) was generated continuously by reacting 7 M H_2_O_2_ with 6.5 M HBr, each fed to a Y-type micromixer and then into a 0.6 mm i.d. coil reactor operated at the set residence time. To handle the substantial exotherm and prevent gas-bubble formation, the mixer and coil were immersed in an ice–water bath before the stream entered the test module.

For bromination, a DCE solution containing 0.6 M EMMA was fed to the test module. The solvents of the bromination reagents (water and DCE) served as reference streams; 1 M Na_2_S_2_O_3_ was used for quenching. Two thermostatic blocks were set to the reaction temperature, and DCE was used to purge the system. Reagents were supplied to both the reaction and reference microreactor chips. After the reaction commenced and signals had stabilised, the calorimetry program was started. Each experimental condition was repeated five times, and the results were averaged. The quenched samples were analysed by HPLC to quantify EMMA (A), monobromide (B), and dibromide (C). Conversion and selectivity were calculated from the HPLC-determined concentrations. These HPLC measurements provide the reference composition data for kinetic parameter estimation and for validating the calorimetry-informed predictions.

### HPLC analysis

HPLC analyses were performed on an Agilent 1260 Infinity II system equipped with a UV diode-array detection and a 4 µm EC-C18 column at 40 °C. The mobile phase (1.0 mL·min^-1^) was 55% (v/v) water and 45% (v/v) acetonitrile, and detection was carried out at 254 nm.

## Supplementary information

Supporting Information

Description of Additional Supplementary Files

Supplementary Movie 1

## Data Availability

All data generated or analysed during this study are included in this published article (and its supplementary information files). The original raw experimental data are stored by the corresponding author and are available from Shengyang Tao upon reasonable request.

## Code availability

The custom Python supervisory program and associated scripts used to implement the calorimetry-informed visual digital model are available from the corresponding author upon reasonable request for academic research purposes.
