# Supplementary material for: Calorimetry informed visual digital model for continuous flow photobromination
Source: Commun Chem. 2026 Apr 11;9:206. doi: 10.1038/s42004-026-02023-5 (PMC13266058; doi:10.1038/s42004-026-02023-5)
Supplement: Supplementary file 1 — Supporting Information [file 42004_2026_2023_MOESM1_ESM.pdf]

Supplementary Information

Calorimetry Informed Visual Digital Model for Continuous Flow Photobromination

Yiming Xu (徐一鸣), Yun Zou (邹昀), Junfei Zhang (张骏飞), Fujun Li (李富军), Shengyang Tao (陶胜洋)*

State Key Laboratory of Fine Chemicals, Frontier Science Center for Smart Materials, School of Chemistry, Dalian University of Technology, Dalian, China

*Correspondence email: taosy@dlut.edu.cn

These authors contributed equally: Yiming Xu, Yun Zou, Junfei Zhang.

Content

1. Experimental point design 1
2. Correlation matrix and predictive-variance calculations 4
3. Single-point deletion and leverage analysis 5
4. Experimental data 6
5. Quadratic response-surface regression and statistics 9
6. 3d isosurface plotting methods and parameters 13
7. Derivation of kinetic formulas 14
8. Workflow of the visual digital model 19

Supplementary Note 1. Experimental point design

We performed a response-surface design over three factors—temperature (*T*), residence time (τ) and light intensity (*I*)—for a total of 36 runs. Each factor was coded to the interval [−1, 1] by linearly mapping its natural value to a dimensionless coded value (−1 = lower bound, 0 = centre, +1 = upper bound).

A candidate set of operating points was constructed within the admissible region ($4 \times 5 \times 4 = 80$), and a D-optimal criterion (quadratic model with 10 regressors: 3 main, 3 squared, 3 pairwise interactions, and an intercept) was used to select 36 operating conditions from the candidate set. Five replicates were placed at the engineering centre point (coded values $T_c = 0.333$, $\tau_c = 0$, $I_c = 0.292$, $T = 40$ °C, $\tau = 80$ s, $I = 35.0$ mW/cm^2) for pure-error estimation and platform-drift monitoring (Supplementary Table S1). Four points with I_c in the low–medium range were included to enhance identifiability of the quadratic curvature in the *I* dimension, and single points at selected axial locations probed second-order curvature and assisted interaction-term estimation along the respective axes.

Supplementary Table 1 36 sets of D-optimal operating points (raw values and coded values listed side-by-side)

Run	T_c	τ_c	I_c	*T* (°C)	τ (s)	*I* (mW/cm^2)
1	1.000	1.000	1.000	45	120	49.2
2	-1.000	-1.000	-1.000	30	40	9.1
3	1.000	-1.000	1.000	45	40	49.2
4	-1.000	-0.500	-1.000	30	60	9.1
5	1.000	1.000	-0.332	45	120	22.5
6	0.333	0.000	0.292	40	80	35.0
7	-1.000	1.000	-1.000	30	120	9.1
8	1.000	0.000	1.000	45	80	49.2

Run	T_c	τ_c	I_c	T (°C)	τ (s)	I (mW/cm^2)
9	-1.000	-1.000	0.292	30	40	35.0
10	-1.000	-1.000	1.000	30	40	49.2
11	1.000	0.500	1.000	45	100	49.2
12	0.333	0.000	0.292	40	80	35.0
13	-0.333	0.000	-0.332	35	80	22.5
14	-1.000	0.000	1.000	30	80	49.2
15	1.000	-1.000	0.292	45	40	35.0
16	1.000	-1.000	-1.000	45	40	9.1
17	1.000	0.000	-1.000	45	80	9.1
18	0.333	0.000	0.292	40	80	35.0
19	-1.000	1.000	1.000	30	120	49.2
20	-1.000	1.000	0.292	30	120	35.0
21	-0.333	-1.000	-1.000	35	40	9.1
22	1.000	1.000	0.292	45	120	35.0
23	-0.333	1.000	-1.000	35	120	9.1
24	0.333	0.000	0.292	40	80	35.0
25	1.000	1.000	0.292	45	120	35.0
26	-0.333	-1.000	0.292	35	40	35.0
27	0.333	-1.000	1.000	40	40	49.2
28	0.333	0.000	-0.332	40	80	22.5
29	0.333	1.000	1.000	40	120	49.2
30	0.333	0.000	0.292	40	80	35.0
31	-0.333	-0.500	-1.000	35	60	9.1
32	-0.333	-1.000	1.000	35	40	49.2
33	0.333	-1.000	-0.332	40	40	22.5
34	-1.000	-0.500	0.292	30	60	35.0
35	1.000	-0.500	0.292	45	60	35.0

Run	T_c	τ_c	I_c	T (℃)	τ (s)	I (mW/cm^2)
36	-0.333	0.500	0.292	35	100	35.0

This design covers low/medium/high levels for each factor, enabling a full quadratic model while centre-point replicates improve reliability and precision.

Supplementary Note 2. Correlation matrix and predictive-variance calculations

We fitted a quadratic response-surface model with 10 basis functions using coded variables: intercept 1; main effects (T_c, τ_c, I_c); squared terms (T_c^2, τ_c^2, I_c^2); interactions $(T_c\tau_c, T_cI_c, \tau_cI_c)$. The design matrix is denoted $\mathbf{X} \in \mathbb{R}^{n\times p}$ (here $n = 36$ runs, $p = 10$ regressors). The weight matrix is $\mathbf{W} = \mathbf{I}$ (identity) absent heteroscedasticity, and the information matrix is

$$\mathbf{M} = \mathbf{X}^{\mathrm{T}}\mathbf{W}\mathbf{X} \tag{S1}$$

For any point with basis vector $f(x)$, the normalised predictive variance is

$$v(x) = f(x)^{\mathrm{T}}\mathbf{M}^{-1}f(x) \tag{S2}$$

proportional to the variance of the fitted response (up to the residual variance factor). The D-efficiency is

$$D_{\text{eff}} = (\frac{\det(\mathbf{M})}{n^p})^{1/p} \tag{S3}$$

with $0 \le D_{\text{eff}} \le 1$. Because quadratic models include squared terms, perfect orthogonality is unattainable; here $D_{\text{eff}} \approx 0.374$ (≈ 37.4%). The 2-norm condition number of $\mathbf{X}$,

$$\kappa_2(\mathbf{X}) = \frac{\sigma_{\max}(\mathbf{X})}{\sigma_{\min}(\mathbf{X})} \tag{S4}$$

is ≈ 3.42, indicating good numerical conditioning and no severe multicollinearity.

Supplementary Note 3. Single-point deletion and leverage analysis

To evaluate how each experimental run influences the model fit, we combined leverage analysis with single-point deletion diagnostics. Leverage is defined as the diagonal element h_i of the hat matrix $\mathbf{H}$,

$$\mathbf{H} = \mathbf{X}(\mathbf{X}^\top\mathbf{X})^{-1}\mathbf{X}^\top \qquad (S5)$$

where $\mathbf{X}$ is the design matrix. Using this design matrix, the leverage of run i is

$$h_i = f(\mathbf{x}_i)^\top(\mathbf{X}^\top\mathbf{X})^{-1}f(\mathbf{x}_i) \qquad (S6)$$

with $f(\mathbf{x}_i)$ the vector of basis functions corresponding to the i-th experiment (i.e., the i-th row of $\mathbf{X}$). The theoretical mean leverage equals p/n, where p is the number of regression terms and n is the number of runs. In this study $p/n = 10/36 \approx 0.28$. A run with h_i substantially higher than this mean is considered a high-leverage point, implying a disproportionate influence on prediction. In our design, no leverage exceeds twice the mean; the maximum leverage is about 0.50. This indicates the absence of abnormally high-leverage runs and a generally balanced influence of individual runs on the fitted model.

In addition, we performed single-point deletion diagnostics (leave-one-out refitting). Specifically, each of the 36 runs was removed from the dataset in turn, the regression model was re-estimated, and changes in model coefficients and predictive performance were examined. If removing a run had caused pronounced shifts in the parameter estimates or a clear increase in prediction error, that run would be deemed influential and potentially anomalous. The results show that deleting any single run leads to changes within acceptable bounds; no model failure or drastic coefficient instability was observed. This agrees with the leverage analysis: the present experimental design contains no run with disproportionate impact on the model. Therefore, the 36 selected experiments contribute information in a balanced manner, yielding stable estimates of the model parameters.

Supplementary Note 4. Experimental data

Supplementary Table 2 Experimental data for the 36 D-optimal runs. Here conversion X and selectivity S are reported in %, and total heat Q in J.

Run	*T* (℃)	*τ* (s)	*I* (mW/cm^2)	*X*	*S*	*Q*(J)
1	45	120	49.2	76.41%	90.64%	18.5421
2	30	40	9.1	17.43%	98.84%	3.7817
3	45	40	49.2	60.83%	89.20%	12.2238
4	30	60	9.1	30.96%	96.28%	5.2542
5	45	120	22.5	72.17%	91.72%	16.4781
6	40	80	35.0	67.96%	94.08%	12.7474
7	30	120	9.1	43.86%	80.14%	8.5644
8	45	80	49.2	71.44%	90.42%	16.3961
9	30	40	35.0	38.46%	88.10%	6.4130
10	30	40	49.2	41.64%	96.03%	7.2343
11	45	100	49.2	72.70%	85.23%	17.6134
12	40	80	35.0	67.98%	94.00%	13.3332
13	35	80	22.5	56.78%	82.36%	10.5223
14	30	80	49.2	58.47%	91.25%	11.0445
15	45	40	35.0	57.99%	95.19%	11.1826
16	45	40	9.1	41.68%	87.48%	7.3516
17	45	80	9.1	58.37%	79.21%	11.3011
18	40	80	35.0	67.74%	93.95%	13.6116
19	30	120	49.2	69.26%	88.81%	13.4057
20	30	120	35.0	63.17%	83.49%	12.4163
21	35	40	9.1	17.48%	96.28%	4.8177
22	45	120	35.0	73.55%	84.91%	17.6752
23	35	120	9.1	54.89%	93.85%	10.2451
24	40	80	35.0	67.95%	93.89%	14.3194

Run	T (°C)	τ (s)	I (mW/cm^2)	X	S	Q(J)
25	45	120	35.0	69.09%	92.64%	13.7955
26	35	40	35.0	43.58%	93.08%	7.8881
27	40	40	49.2	55.48%	84.65%	10.4793
28	40	80	22.5	61.15%	94.40%	12.2873
29	40	120	49.2	72.54%	88.79%	16.8932
30	40	80	35.0	67.65%	93.75%	14.3391
31	35	60	9.1	39.13%	84.45%	6.5430
32	35	40	49.2	53.56%	95.69%	8.8011
33	40	40	22.5	41.69%	95.21%	7.5866
34	30	60	35.0	43.60%	88.34%	8.4580
35	45	60	35.0	66.55%	91.89%	13.6796
36	35	100	35.0	64.27%	86.92%	13.1415

NMR and HRMS for product

methyl (*E*)-2-(2-(bromomethyl)phenyl)-2-(methoxyimino)acetate[1]

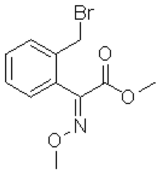

^{1}H NMR (400 MHz, $CDCl_3$) δ 7.51 – 7.47 (m, 1H), 7.44 – 7.40 (m, 1H), 7.39 – 7.34 (m, 1H), 7.18 – 7.13 (m, 1H), 4.34 (s, 2H), 4.07 (s, 3H), 3.88 (s, 3H).

HRMS (ESI) calculated for $C_{11}H_{13}BrNO_3$ [M+H$^+$]: 286.0073, found: 286.0070.

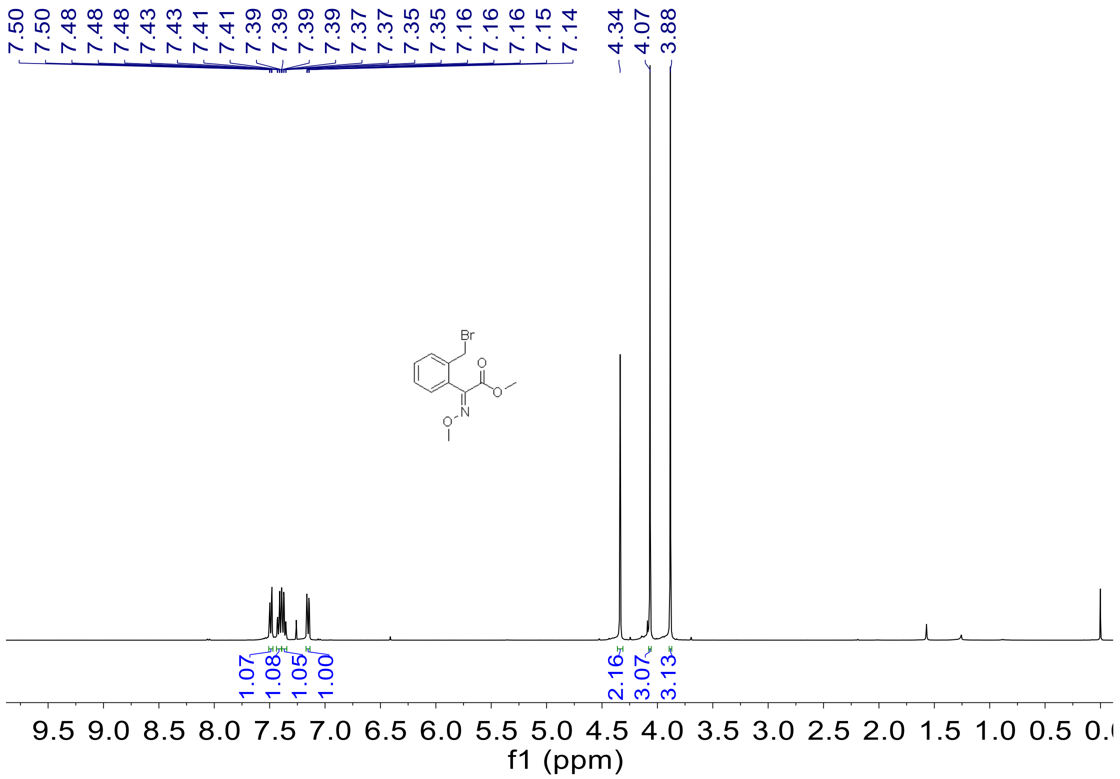
7.50
7.50
7.48
7.48
7.48
7.43
7.43
7.41
7.41
7.39
7.39
7.39
7.39
7.37
7.37
7.35
7.35
7.16
7.16
7.16
7.15
7.14
4.34
4.07
3.88
Br
O
O
N
O
1.07
1.08
1.05
1.00
2.16
3.07
3.13
9.5 9.0 8.5 8.0 7.5 7.0 6.5 6.0 5.5 5.0 4.5 4.0 3.5 3.0 2.5 2.0 1.5 1.0 0.5
f1 (ppm)

Supplementary Note 5. Quadratic response-surface regression and statistics

For each response—conversion X, selectivity S, and total heat Q—we fitted an ordinary least-squares (OLS) quadratic model. Let $\hat{y} = \mathbf{X}\hat{\beta}$ be the fitted value, with coefficient vector $\hat{\beta} = (\mathbf{X}^{\mathrm{T}}\mathbf{X})^{-1}\mathbf{X}^{\mathrm{T}}y$. Goodness-of-fit metrics are

$$R^2 = 1 - \frac{\sum_{i=1}^{n}(y_i - \hat{y}_i)^2}{\sum_{i=1}^{n}(y_i - \bar{y})^2} \tag{S7}$$

$$\mathrm{RMSE} = \sqrt{\frac{1}{n}\sum_{i=1}^{n}(y_i - \hat{y}_i)^2} \tag{S8}$$

with sample size $n = 36$ and regressor count $p = 10$. (Here y is the response vector; $\bar{y}$ its mean.)

Conversion X

Main effects of T, τ, I are positive and significant; squared and interaction terms indicate diminishing returns and coupling attenuation.

Supplementary Table 3. Conversion model for X (standardised variables).

Term	Estimate	*p*-value
Intercept	0.6293	< 0.0001
T	0.0631	< 0.0001
τ	0.0918	< 0.0001
I	0.0640	< 0.0001
T^2	−0.0076	0.2640
τ^2	−0.0361	< 0.0001
I^2	−0.0177	0.0051
Tτ	−0.0148	0.0023
TI	−0.0127	0.0091
τI	−0.0125	0.0089

Selectivity S

The overall fit is limited; only τ shows a weak negative correlation.

Supplementary Table 4. Selectivity model for S (standardised variables).

Term	Estimate	*p*-value
Intercept	0.9160	$< 1.0\times10^{-20}$
T	−0.0053	0.541
τ	−0.0182	0.043
I	0.0020	0.831
T^2	−0.0129	0.283
τ^2	0.0071	0.529
I^2	−0.0083	0.426
$T\tau$	0.0109	0.173
TI	0.0052	0.520
τI	0.0057	0.473

Total heat Q

Main effects of T, τ, I are positive and significant; τ^2 is significantly negative.

Supplementary Table 5. Heat model for Q (standardised variables).

Term	Estimate	*p*-value
Intercept	12.373	$< 1.0\times10^{-20}$
T	1.791	3.77×10^{-13}
τ	2.376	4.93×10^{-16}
I	1.579	2.66×10^{-11}
T^2	−0.0311	0.867
τ^2	−0.682	5.52×10^{-4}
I^2	−0.282	0.091
$T\tau$	−0.0201	0.870
TI	0.109	0.390

τI	0.0795	0.524

Model parsimony and hierarchical term screening

The full quadratic response surface above was adopted as the candidate basis for the D-optimal design and the initial response surface regression. After data acquisition, we revisited the regression to obtain parsimonious surrogate models for each response. Term screening started from the full basis set consisting of T, τ, I, T^2, τ^2, I^2, $T\tau$, TI and τI.

We applied hierarchical backward elimination to second order terms and removed terms with p value larger than 0.05 while preserving model hierarchy. Whenever a second order term was retained, its associated main effects were kept in the model. Model adequacy was evaluated by R^2 and adjusted R^2 together with prediction error metrics including the training RMSE and the leave-one-out (LOO) RMSE. LOO RMSE was computed from the PRESS residuals $e_i/(1 - h_{ii})$, where e_i is the OLS residual and h_{ii} is the diagonal element of the hat matrix.

For conversion X, only the T^2 term was removed, and all remaining terms were statistically supported. For selectivity S, the reduced model retains only τ in addition to the intercept, consistent with the limited variance of S within the studied window. For total heat Q, the interaction terms and the T^2 and I^2 terms were not supported and were removed, leaving T, τ, I and τ^2 as the retained predictors. The performance comparison between the full and reduced models is summarised in Supplementary Table S6.

Supplementary Table 6. Summary of hierarchical term screening and predictive performance for the quadratic surrogate models. RMSE and LOO RMSE are reported as percentage points for *X* and *S*, and in *J* for *Q*.

Response	Model	Retained terms	R^2	Adj R^2	RMSE	LOO RMSE
X	Full	Intercept, T, τ, I, T^2, τ^2, I^2, $T\tau$, TI, τI	0.977	0.969	2.32	3.41
X	Reduced	Intercept, T, τ, I, τ^2, I^2, $T\tau$, TI, τI	0.976	0.968	2.38	3.34
S	Full	Intercept, T, τ, I, T^2, τ^2, I^2, $T\tau$, TI, τI	0.285	0.037	4.13	6.04
S	Reduced	Intercept, τ	0.126	0.100	4.57	4.82
Q	Full	Intercept, T, τ, I, T^2, τ^2, I^2, $T\tau$, TI, τI	0.972	0.962	0.65	0.86
Q	Reduced	Intercept, T, τ, I, τ^2	0.968	0.964	0.69	0.81

Supplementary Note 6. 3D isosurface plotting methods and parameters

For reproducibility, each raw predictor $x \in \{T, \tau, I\}$ was standardised as

$$x^* = \frac{x - \mu}{\sigma} \qquad (S9)$$

where μ is the sample mean and σ the standard deviation used for plotting (Supplementary Table S7). A dense grid over the operating domain produced predictions $\hat{y}(x)$; isosurfaces are level sets $\hat{y}(x) = c$ at the median (p50), upper-quartile (p75) and top-decile (p90) of $\hat{y}$. The 36 experimental points are overlaid in T–τ–I space to assess coverage and extrapolation risk.

Supplementary Table 7. Statistics for input variables used in 3D plotting.

Variable	μ	σ
T (°C)	38.06	5.81
τ (s)	76.67	30.73
I (mW/cm^2)	31.80	14.57

Supplementary Note 7. Derivation of kinetic formulas

We adopt a two-step consecutive scheme: A (EMMA) $\overset{k_1}{\to}$B (monobromide) $\overset{k_2}{\to}$C (dibromide). Let $[A],[B],[C]$ be molar concentrations (mol/L); apparent rate constants k_1,k_2 have units s^{-1}; light intensity I (mW/cm^2) enters via intensity exponents m_1,m_2 (dimensionless). Assuming a plug-flow reactor (PFR), isothermal operation at set T, constant volumetric flow and negligible axial dispersion:

$$\frac{d[A]}{d\tau} = -k_1 I^{m_1}[A] \qquad (S10)$$

$$\frac{d[B]}{d\tau} = k_1 I^{m_1}[A] - k_2 I^{m_2}[B] \qquad (S11)$$

$$\frac{d[C]}{d\tau} = k_2 I^{m_2}[B] \qquad (S12)$$

with inlet $= A_0, == 0$. Solutions:

$$[A](\tau) = A_0\, e^{-k_1 I^{m_1}\tau} \qquad (S13)$$

$$[B](\tau) = \frac{k_1 I^{m_1}}{k_2 I^{m_2} - k_1 I^{m_1}}\, A_0 (e^{-k_1 I^{m_1}\tau} - e^{-k_2 I^{m_2}\tau}) \qquad (S14)$$

$$[C](\tau) = A_0 (1 - \frac{k_2 I^{m_2}\, e^{-k_1 I^{m_1}\tau} - k_1 I^{m_1}\, e^{-k_2 I^{m_2}\tau}}{k_2 I^{m_2} - k_1 I^{m_1}}) \qquad (S15)$$

Define conversion $X = 1 - [A](\tau)/A_0$ and selectivity to B

$$S = \frac{[B](\tau)}{A_0 - [A](\tau)} \qquad (S16)$$

The Arrhenius–power form for temperature/intensity dependence is

$$k_j(T,I) = A_j \exp(-\frac{E_j}{RT_{\mathrm{K}}})\, I^{m_j} (j = 1,2) \qquad (S17)$$

where A_j is the pre-exponential factor (s^{-1}·(mW·cm^{-2})^{−m$_i$}), E_j the activation energy (J/mol), R the gas constant (8.314 J/mol · K), and T_{K} the absolute temperature (K).

Let ΔH_1 and ΔH_2 be reaction enthalpies (kJ/mol, exothermic negative). Under the plug-flow assumption, concentrations and hence reaction rates vary along the residence-time coordinate t. The local volumetric heat-release rate is $q'''(t) = (-\Delta H_1)\, r_1(t) + (-\Delta H_2)\, r_2(t)$, with $r_1(t) = k_1 I^{m_1} C_A(t)$ and $r_2(t) = k_2 I^{m_2} C_B(t)$. The reactor-level heat-release power measured by the DTRCC corresponds to the

spatial integral of the local heat-release rate over the reacting volume, which can be written as

$$P = \int_0^{V_{\text{eff}}} q''' \, dV = \dot{V} \int_0^{\tau} [(-\Delta H_1)\, r_1(t) + (-\Delta H_2)\, r_2(t)]\, dt \qquad \text{(S18)}$$

where $\dot{V}$ is the total volumetric flow rate and $\tau = V_{\text{eff}}/\dot{V}$ is the residence time. The concentration profiles $C_A(t)$and $C_B(t)$are obtained by solving the plug-flow material-balance differential equations in Eq. (S14)–(S16). Fitting yields $\Delta H_1 = -293.9$ kJ/mol and $\Delta H_2 = -113.8$ kJ/mol, the model reproduces total heat with $R^2 = 0.957$, conversion with $R^2 = 0.894$, and mean absolute error in selectivity ≈ 4.15%. Within the studied window, step 2 shows negligible *I*-dependence, suggesting thermal control.

Steady state outlet samples were quenched and analysed by HPLC to quantify the absolute concentrations of EMMA (A), monobromide (B), and dibromide (C). The conversion X and the monobromide selectivity S reported in Supplementary Table S2 were calculated from these HPLC determined concentrations. To directly validate the kinetic model at the composition level, we compared the model predicted outlet concentrations with the experimentally determined HPLC concentrations across the full D-optimal dataset.

For each operating point, the outlet composition predicted by the kinetic model was obtained by evaluating the PFR material balances in Eqs. (S14)–(S16) at the residence time τ using the corresponding operating inputs (*T*, *τ*, *I*). The associated goodness of fit and error statistics are summarised in Supplementary Table S8. This comparison demonstrates that the kinetic model reproduces the experimentally measured outlet composition, providing a robust basis for the subsequent calorimetry-informed visual digital model.

Supplementary Table 8. Error statistics for kinetic model predictions of HPLC outlet concentrations (n = 36).

Species	MAE (mol/L)	RMSE (mol/L)	Max |error| (mol/L)
A (EMMA)	0.0262	0.0322	0.0739

B (monobromide)	0.0210	0.0244	0.0530
C (dibromide)	0.0123	0.0160	0.0385

Supplementary Table 9. Comparison of HPLC-determined outlet concentrations and kinetic-model predictions for each DoE run.

Run	$C_{A,HPLC}$ (mol/L)	$C_{A,model}$ (mol/L)	$C_{B,HPLC}$ (mol/L)	$C_{B,model}$ (mol/L)	$C_{C,HPLC}$ (mol/L)	$C_{C,model}$ (mol/L)
1	0.1056	0.0494	0.3101	0.3318	0.0320	0.0667
2	0.3697	0.3531	0.0771	0.0909	0.0009	0.0038
3	0.1754	0.2147	0.2430	0.2214	0.0294	0.0117
4	0.3092	0.3135	0.1335	0.1260	0.0052	0.0082
5	0.1246	0.0871	0.2964	0.3062	0.0268	0.0544
6	0.1435	0.1485	0.2863	0.2697	0.0180	0.0296
7	0.2514	0.2195	0.1574	0.2000	0.0390	0.0283
8	0.1279	0.1030	0.2893	0.3080	0.0306	0.0368
9	0.2756	0.3011	0.1517	0.1401	0.0205	0.0066
10	0.2613	0.2850	0.1791	0.1552	0.0074	0.0076
11	0.1222	0.0713	0.2775	0.3250	0.0481	0.0515
12	0.1434	0.1485	0.2861	0.2697	0.0183	0.0296
13	0.1935	0.2026	0.2094	0.2228	0.0449	0.0225
14	0.1860	0.1814	0.2389	0.2407	0.0229	0.0257
15	0.1881	0.2348	0.2472	0.2027	0.0125	0.0103
16	0.2612	0.3043	0.1633	0.1374	0.0234	0.0062
17	0.1864	0.2067	0.2070	0.2198	0.0543	0.0213
18	0.1445	0.1485	0.2850	0.2697	0.0184	0.0296
19	0.1377	0.1154	0.2754	0.2830	0.0347	0.0494
20	0.1649	0.1361	0.2362	0.2672	0.0467	0.0445
21	0.3695	0.3381	0.0754	0.1051	0.0029	0.0045
22	0.1184	0.0646	0.2797	0.3220	0.0497	0.0612
23	0.2020	0.1928	0.2307	0.2225	0.0151	0.0325
24	0.1435	0.1485	0.2857	0.2697	0.0186	0.0296
25	0.1384	0.0646	0.2866	0.3220	0.0228	0.0612
26	0.2526	0.2801	0.1816	0.1600	0.0135	0.0078
27	0.1994	0.2389	0.2103	0.1987	0.0381	0.0102
28	0.1740	0.1762	0.2585	0.2460	0.0153	0.0256
29	0.1230	0.0680	0.2884	0.3188	0.0364	0.0610
30	0.1449	0.1485	0.2840	0.2697	0.0189	0.0296
31	0.2726	0.2938	0.1480	0.1443	0.0272	0.0096
32	0.2080	0.2624	0.2295	0.1765	0.0103	0.0088
33	0.2611	0.2809	0.1777	0.1593	0.0089	0.0076

34	0.2526	0.2469	0.1725	0.1871	0.0228	0.0138
35	0.1498	0.1700	0.2738	0.2570	0.0242	0.0208
36	0.1600	0.1385	0.2502	0.2716	0.0376	0.0376

To quantify uncertainty in the fitted kinetic parameters, we evaluated the asymptotic covariance matrix of the nonlinear least-squares estimator using the Gauss–Newton approximation. The Jacobian was computed for the outlet-concentration predictions of A, B and C across the full D-optimal dataset (n = 36 operating points, N = 108 concentration observations). The covariance matrix was approximated as $\mathrm{cov}(\hat{\theta}) = s^2(J^TJ)^{-1}$, where $s^2 = \mathrm{SSE}/(N-p)$ and p is the number of fitted parameters. Two-sided 95% confidence intervals were calculated as $\hat{\theta} \pm t_{0.975,N-p}\,\mathrm{SE}(\hat{\theta})$. For the pre-exponential factors *A*1 and *A*2, confidence intervals are reported on a log scale and back-transformed to enforce positive bounds.

Because Arrhenius parameters are known to exhibit strong correlation between the pre-exponential factor and activation energy, especially over narrow temperature windows, we also report an equivalent reference-temperature parameterisation. Defining $k_{i,\mathrm{ref}} = k_i(T_{\mathrm{ref}}, I_{\mathrm{ref}})$ at T_{ref} = 38.1 °C and I_{ref} = 31.8 mW·cm^{-2}, the rate constants can be written as $k_i(T,I) = k_{i,\mathrm{ref}}\ (I/\ I_{\mathrm{ref}})$^$mi$ $\exp[-E_i/R\ (1/T-1/T_{\mathrm{ref}})]$, which reduces collinearity between the fitted magnitude and E_i.

Supplementary Table 10. Kinetic parameter estimates with 95% confidence intervals.

Parameter	Estimate	95% confidence interval	Unit
*A*1	77.36	5.74–1.04×10^{3}	s^{-1}·(mW·cm^{-2})^(-m1)
*m*1	0.38	0.30–0.46	–
*E*1	26	19.38–32.62	kJ·mol^{-1}
*A*2	3.42×10^{-3}	1.57×10^{-8}–745.62	s^{-1}·(mW·cm^{-2})^(-m2)
*m*2	0.064	-0.43–0.56	–
*E*2	1.7	-29.31–32.71	kJ·mol^{-1}

$k_{1,\mathrm{ref}}$ (T_{ref}=38.1 °C, I_{ref}=31.8 mW·cm^{-2})	0.012	0.012–0.013	s^{-1}
$k_{2,\mathrm{ref}}$ (T_{ref}=38.1 °C, I_{ref}=31.8 mW·cm^{-2})	2.21×10^{-3}	1.69×10^{-3}–2.73×10^{-3}	s^{-1}

Supplementary Table 11. Correlation matrix for fitted kinetic parameters.

	A1	m1	E1	A2	m2	E2
A1	1.000	-0.212	0.995	0.296	-0.075	0.294
m1	-0.212	1.000	-0.110	-0.099	0.324	-0.055
E1	0.995	-0.110	1.000	0.291	-0.041	0.295
A2	0.296	-0.099	0.291	1.000	-0.294	0.990
m2	-0.075	0.324	-0.041	-0.294	1.000	-0.156
E2	0.294	-0.055	0.295	0.990	-0.156	1.000

Supplementary Table 12. Correlation matrix in the reference-temperature parameterisation ($k_{i,\mathrm{ref}}$, m_i, E_i).

	k1,ref	m1	E1	k2,ref	m2	E2
k1,ref	1.000	0.169	-0.035	0.308	-0.022	-0.090
m1	0.169	1.000	-0.110	-0.020	0.324	-0.055
E1	-0.035	-0.110	1.000	-0.083	-0.041	0.295
k2,ref	0.308	-0.020	-0.083	1.000	-0.154	-0.369
m2	-0.022	0.324	-0.041	-0.154	1.000	-0.156
E2	-0.090	-0.055	0.295	-0.369	-0.156	1.000

Supplementary Note 8. Workflow of the visual digital model

The system implements a closed-loop workflow—a-priori prediction → calorimetry → selectivity correction → refreshed outputs—to realize a visual, digital twin for continuous-flow photochemistry. The user first inputs the reactor geometry and volume, feed concentrations, total volumetric flow rate, set temperature, and light-intensity information. The system performs unit checking and couples these inputs to the model.

The software–hardware interaction architecture underlying this workflow is summarised in Supplementary Figure S1. A Python-based supervisory program runs on the host computer and coordinates two synchronized hardware chains: an RS-485/Modbus RTU chain for six-channel syringe-pump control, and a DAQ/PWM-DAC chain for calorimetric signal acquisition and power-compensation control. These two chains are synchronized by shared experiment-state flags and unified timestamps, enabling closed-loop execution of the online prediction–measurement–update workflow. In practice, the program first generates a kinetics-based prior prediction from the user-specified operating conditions (T, τ, I), then acquires the calorimetric heat-release signal, and finally performs a calorimetry-informed posterior correction to update the displayed reactor maps and thermal-management guidance.

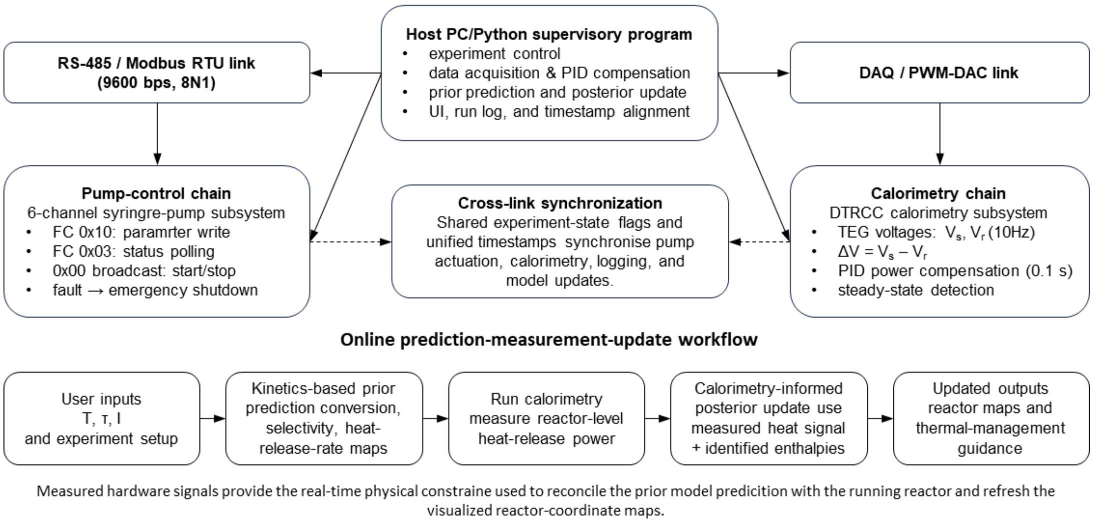

Supplementary Figure 1 Software–hardware interaction architecture and online

visualisation workflow.

For the model calculations shown in Supplementary Figure S1, the microreactor is abstracted as a two-dimensional computational domain Ω. Radial concentration gradients are neglected, and the local process state is described in a Lagrangian "residence-time" coordinate. The equivalent residence-time field $\tau(x,y)$ is obtained from a steady advection problem,

$$\mathbf{u}(x,y)\cdot\nabla\tau(x,y)=1,\tau\mid_{\Gamma_{\text{in}}}=0 \tag{S19}$$

where $\mathbf{u}(x,y)$ is the velocity field determined by the reactor geometry and the total flow rate.

Along a streamline, the species balances for the two-step network $A\to B\to C$ read

$$\frac{dc_A}{dt}=-r_1,\frac{dc_B}{dt}=r_1-r_2,\frac{dc_C}{dt}=r_2 \tag{S20}$$

where c_i are concentrations and r_1,r_2 are the reaction rates predicted by the a-priori kinetics. With reaction enthalpies $\Delta H_1,\Delta H_2$ (negative for exothermic reactions), the local volumetric heat-release rate is

$$\dot{q}(x,y)=-\Delta H_1\,r_1(x,y)-\Delta H_2\,r_2(x,y) \tag{S21}$$

The reactor-level heat-release power is obtained by spatial integration,

$$\dot{Q}_{\text{pred}}(t)=\int_{\Omega}\dot{q}(x,y,t)\,d\Omega \tag{S22}$$

During the a-priori stage the first set of 2D maps (conversion, selectivity, and heat-release rate) is generated. At the same time, the predicted heat load (heat-release power) is used to give an initial thermal-management recommendation. For an allowable water-side temperature rise $\Delta T_{w,\text{allow}}$, the required cooling-water mass flow rate is

$$\dot{m}_w(t)=\frac{\dot{Q}(t)}{c_{p,w}\,\Delta T_{w,\text{allow}}} \tag{S23}$$

Consistency is checked against a pre-calibrated overall heat-transfer conductance UA via the log-mean temperature difference (LMTD) method,

$$\dot{Q}(t) = UA\,\Delta T_{\mathrm{lm}}(t), \Delta T_{\mathrm{lm}} = \frac{\Delta T_{\mathrm{in}} - \Delta T_{\mathrm{out}}}{\ln(\Delta T_{\mathrm{in}}/\Delta T_{\mathrm{out}})} \qquad (S24)$$

where $\Delta T_{\mathrm{in}}$and ΔT_{out} are the inlet and outlet temperature approaches between the process stream and coolant.

After the baseline becomes stable, the calorimetry stage starts. A combined slope- and moving-variance criterion is used to detect steady segments for data acquisition; empirically, one operating condition is completed in $\sim$150 s, yielding the measured calorimetric heat-release power trace $P_{\mathrm{meas}}(t)$. The reported heat metric for the DoE dataset is the steady-state mean power $\bar{P}_{\mathrm{meas}}$ converted to the total heat released over the residence time, $Q = \bar{P}_{\mathrm{meas}}\tau$, highlighting that Q is an integral quantity whereas P_{meas} is a reactor-level heat-flow (power) signal. In the second prediction, only the selectivity is corrected while keeping the previously predicted overall rate. Define the total rate $r_{\mathrm{tot}} = r_1 + r_2$and the branching fraction $\rho = r_2/r_{\mathrm{tot}}$. Let $h_i = -\Delta H_i > 0$ denote the molar heat release. At each instant (or over a short time window),

$$\dot{Q}_{\mathrm{pred}}(\rho) = \int_{\Omega} [h_1(1-\rho) + h_2\rho]\, r_{\mathrm{tot}}\, d\Omega = H_0 + \rho\, H_{\Delta} \qquad (S25)$$

with $H_0 = \int_{\Omega} h_1 r_{\mathrm{tot}}\, d\Omega$ and $H_{\Delta} = \int_{\Omega}(h_2 - h_1) r_{\mathrm{tot}}\, d\Omega$, both available from the a-priori prediction. Aligning $\dot{Q}_{\mathrm{pred}}(\rho)$ with $\dot{Q}_{\mathrm{meas}}$ gives a closed-form correction for the branching fraction,

$$\rho^* = \frac{\dot{Q}_{\mathrm{meas}} - H_0}{H_{\Delta}} \qquad (S26)$$

subject to the physical bound $0 \le \rho^* \le 1$ and mild temporal smoothing to suppress noise. Keeping r_{tot} unchanged, the corrected stepwise rates are

$$r_1^{\mathrm{corr}} = (1-\rho^*)\, r_{\mathrm{tot}}, r_2^{\mathrm{corr}} = \rho^*\, r_{\mathrm{tot}} \qquad (S27)$$

Material balances and heat-release calculations are then re-integrated to refresh the 2D maps of selectivity and heat-release rate, and a second thermal-management recommendation is issued accordingly. The conversion map is kept from the kinetic a-priori stage, thereby clearly separating “conversion dictated by kinetics” from “selectivity corrected by calorimetry.” Empirically, compared with directly fitting the

heat-release power trace alone, the branching-ratio correction yields 2D maps whose hot-spot locations and overall reaction progress agree substantially better with experimental observations. The final outputs include steady 2D fields and a structured database; predicted $\dot{Q}$ remains consistent with the UA–LMTD check. Whenever operating inputs are updated, the steady flow field, local heat-transfer consistency checks, and all color maps are synchronously refreshed in the interface, enabling traceable iterative operation.

Supplementary References

1 Li, F., Cheng, P., Li, X., Zou, Y. & Tao, S. Continuous-Flow Photobromination/Debromination Cascade for Synthesis of the Trifloxystrobin Intermediate. *Industrial & Engineering Chemistry Research* **63**, 17734-17743, doi:10.1021/acs.iecr.4c02202 (2024).
