# Supplementary material for: Calorimetry informed visual digital model for continuous flow photobromination
Source: Commun Chem. 2026 Apr 11;9:206. doi: 10.1038/s42004-026-02023-5 (PMC13266058; doi:10.1038/s42004-026-02023-5)
Supplement: Supplementary file 2 — Description of Additional Supplementary Files [file 42004_2026_2023_MOESM2_ESM.pdf]

Description of Additional Supplementary Files:

File: Supplementary Movie 1

Description: Visual Digital Modeling System Based on Heat Measurement Data (speed ×15).
